# Management strategies and determinants of clinical decision-making in oroantral communication among dental practitioners

**DOI:** 10.1007/s10006-026-01508-w

**Published:** 2026-01-30

**Authors:** Man Hung, Samantha Lee, Jacob Marx, Corban Ward, Owen Cohen, Madeleine Tucker, Charles Miller

**Affiliations:** 1 College of Dental Medicine, Roseman University of Health Sciences, South Jordan, UT USA; 2 Department of Family and Preventive Medicine, University of Utah, Salt Lake City, UT USA; 3 Department of Educational Psychology, University of Utah, Salt Lake City, UT USA; 4 Huntsman Cancer Institute, Salt Lake City, UT USA

**Keywords:** Oroantral communication, Oroantral fistula, Sinus perforation, Management strategies, Surgical training

## Abstract

**Introduction:**

Oroantral communication (OAC) represents an abnormal connection between the oral cavity and the maxillary sinus, often arising after posterior maxillary extractions. Unmanaged OACs can lead to chronic sinusitis and fistula formation. Despite various treatment options, standardized guidelines are lacking. This study examined current management strategies, influencing factors, and practitioner confidence in treating OACs among dental professionals in the United States (U.S.).

**Methods:**

A nationwide, cross-sectional online survey was conducted among 193 dental practitioners. The survey assessed demographics, management preferences by perforation size, timing of repair, material selection factors, and self-reported comfort levels. Associations between practitioner characteristics and management choices were analyzed using Cramer’s V. Multivariate logistic regressions were also performed to evaluate independent predictors of (1) management choice, and (2) timing of repair.

**Results:**

Most respondents were male (72.8%) and mid-career practitioners. Management approaches varied by perforation size: collagen-based materials were common for small defects (0–2 mm), while specialist referral and flap techniques predominated for larger perforations (> 7 mm). 81% performed closure on the same day. Ease of use was the main factor guiding material choice (33.7%). Specialization showed a strong association with management approach (Cramer’s V = 0.654, *p* < 0.001), and experience with OAC cases was moderately to strongly related (Cramer’s V = 0.403, *p* = 0.007). In multivariate analyses, experience managing sinus perforations and self-rated confidence were associated with management choice, while no practitioner characteristics were independently associated with timing of repair.

**Conclusion:**

Respondents demonstrated size-dependent escalation in OAC management, but variability persisted in technique and material selection. Adjusted analyses suggest that experience-related factors are more closely linked to material selection for moderate defects, whereas timing of repair appears relatively consistent across practitioner characteristics. These findings highlight the need for standardized, outcomes-informed guidance and targeted training to improve consistency and optimize patient outcomes.

## Introduction

Oroantral communication (OAC) refers to a pathological connection between the oral cavity and the maxillary sinus [1]. This bi-directional pathway permits transfer of bacteria, fluids, and debris between compartments, increasing the risk of acute or chronic sinusitis, persistent infection, and progression to an oroantral fistula (OAF), thereby compromising quality of life [2]. OAC most commonly occurs when the maxillary sinus is inadvertently opened during surgical procedures in the posterior maxilla, particularly extraction of maxillary premolars or molars or removal of implants, due to the close anatomic relationship between the sinus floor and posterior root apices [3–6].

While small communications may close spontaneously, persistent defects can epithelialize and form an OAF—an epithelialized tract between the oral cavity and sinus—especially when ongoing passage of air, fluids, microbes, or debris prevents mucosal edge approximation; once epithelialized, spontaneous closure becomes unlikely and the risk of chronic sinusitis or sinus abscess increases [7–9]. Patient- and behavior-related factors (e.g., sinonasal health, immune status, smoking, and nose-blowing) may further reduce the likelihood of spontaneous closure [10]. To reduce fistula formation, expert recommendations generally favor early closure—often within 24–48 h—when clinically feasible, to limit bacterial contamination and re-establish a barrier between the oral cavity and maxillary sinus [4, 11].

OAF formation can present challenges in both diagnosis and treatment, necessitating a comprehensive understanding of current treatment modalities. Given the potential complications arising from sinus communication, many closure techniques have been explored and reported in the literature. As such, there are inconsistencies in the management of sinus communications between general dentists and specialists. Additionally, the development of more treatment techniques can make it more difficult to standardize the management of sinus communications.

Treatment variability extends to the choice of materials and techniques employed for closure. The variability in treatment practices can be attributed to differences in clinical training, experience, and available resources. Management of OACs and OAFs encompasses a range of approaches. For example, multidisciplinary strategies for odontogenic sinusitis associated with OAC/OAF have been reported to achieve symptom resolution and fistula closure [12]. Buccal advancement flap techniques have also demonstrated favorable healing outcomes [13]. The buccal sliding flap remains a popular and simple method for managing OACs [14, 15]. For smaller OACs (≤ 5 mm), platelet-rich fibrin has been reported to support full epithelialization with low complication rates [4]. A one-stage approach combining titanium mesh with functional endoscopic sinus surgery has been described for OACs complicated by chronic sinusitis, offering improved drainage and tissue stability [16]. Techniques incorporating sinus bone grafting with fixation (e.g., bone tacks), a collagen membrane, and allograft have also been described, enabling both closure and bone regeneration for future rehabilitation [5, 6]. Collectively, these options underscore the importance of tailoring management to defect characteristics and the broader clinical diagnosis. Where odontogenic sinusitis is present, interdisciplinary collaboration with otolaryngology may optimize outcomes [17].

Despite numerous proposed techniques, no universally accepted clinical guideline currently exists for OAC management. This lack of consensus can result in variability in patient care and outcomes. In addition, a recent systematic review has summarized the existing clinical literature on OAC/OAF management and generally support size- and sinus-status–based treatment principles; however, it does not describe how these principles are implemented in routine practice across provider types (e.g., referral thresholds, material/technique selection drivers, and practitioner confidence) [10]. Understanding current clinical practices and the factors influencing treatment decisions is critical to developing evidence-based protocols. Treatment selection is influenced by numerous factors, including size and location of the defect, the amount and condition of tissue available for repair, the presence of infection, surgeon competence, and the time elapsed since the onset/diagnosis of the fistula [16]. A multidisciplinary approach, particularly for patients suffering from odontogenic sinusitis associated with OACs, allows for a comprehensive evaluation of each patient’s unique circumstances and the development of a tailored treatment plan [12]. This multidisciplinary approach may involve input from oral and maxillofacial surgeons, otolaryngologists, and other specialists as needed, ensuring that all aspects of the patient’s condition are addressed. This personalized methodology recognizes that each patient presents a distinct set of challenges and requirements, necessitating a flexible and adaptable approach to treatment selection and implementation. In short, individualized, evidence-based care is preferable to a one-size-fits-all algorithm.

The present study aimed to: (1) identify the most common treatment options used by dental practitioners for managing OAC of various sizes, and (2) examine the frequency with which practitioners encounter and manage sinus perforations, and the factors influencing their management strategies. By elucidating real-world decision-making according to defect size, timing, and sinus health, and by evaluating clinician- and practice-level factors associated with key management decisions, this study seeks to identify practice trends, gaps, and opportunities for standardization. These findings may ultimately contribute to the development of clearer clinical guidelines and more effective, patient-centered management strategies, while supporting rehabilitation in the posterior maxilla.

## Methods

### Survey design and setting

We conducted a nationwide, cross-sectional, web-based survey of practicing United States (U.S.) dentists. Following institutional review board (IRB) approval, participants were recruited through a multimodal outreach strategy, including direct telephone invitations to private practices, email distribution via professional and state dental society listservs, and targeted posts in closed professional social media groups. Participation was voluntary, anonymous, and uncompensated. No protected health information or identifiable data were collected to ensure respondent confidentiality and compliance with ethical standards.

### Participants

Eligible respondents included licensed general dentists and dental specialists currently engaged in clinical practice within the U.S. Exclusion criteria encompassed dentists practicing outside the U.S., current trainees (residents or fellows) without independent clinical responsibility, and retired clinicians not involved in active patient care. We employed a convenience sampling approach with a broad geographic and demographic reach to capture a diverse representation of training backgrounds, years of experience, and practice settings. This design aimed to reflect real-world variation in clinical exposure and management strategies for oroantral communications.

### Survey instrument

The survey instrument was a structured, self-administered questionnaire developed based on a comprehensive review of the existing literature on OAC and fistula management. To ensure content validity, the survey was reviewed and refined in consultation with an oral and maxillofacial surgeon (OMS) with expertise in maxillary sinus management, and a psychometrician with expertise in survey development. The final survey was implemented electronically via Google Forms and was available to participants over a 12-month data collection period. Prior to deployment, cognitive pretesting was conducted with a small sample to assess clarity, item comprehension, logical flow, and completion time. Revisions were made accordingly to improve usability and reduce ambiguity. The finalized survey comprised four domains: (1) demographic and practice characteristics, (2) clinical experience with sinus perforations, (3) management practices, and (4) knowledge, confidence, and beliefs. Management choices were captured using structured multiple-choice formats.

### Outcomes and covariates

Primary outcomes were: (a) reported frequency of sinus perforations, (b) immediate management choices by defect size, and (c) preferred definitive closure techniques/materials. Secondary outcomes included timing of closure. Prespecified covariates were age, sex, education, year of practice dentistry specialty, patient volume, extraction volume, confidence, and practice location. These covariates were selected a priori based on clinical relevance and potential influence on treatment selection.

### Data analyses

Survey data were exported from Google Forms into a secure database, cleaned, and coded according to a predefined codebook. Analyses were conducted using Python (version 3.11). Categorical variables were summarized using counts and percentages, while continuous variables were expressed as means with standard deviations or medians with interquartile ranges, as appropriate.

To examine factors associated with key management choices (i.e., buccal advancement flap vs. alternatives) and time point for repair (i.e., immediate closure vs. at a later time), we used Cramer’s V coefficient to assess the strength of association on the prespecified covariates. Cramer’s V ranges from 0 (no association) to 1 (perfect association), and its interpretation followed Rea and Parker’s effect size interpretation: 0.00 to 0.10 for negligible, 0.10 to 0.20 for weak, 0.20 to 0.40 for moderate, 0.40 to 0.60 for relatively strong, 0.60 to 0.80 for strong, and 0.80 to 1.00 for very strong strength of association [18].

To further evaluate independent associations and reduce confounding, we also conducted multivariate logistic regression analyses for two clinically relevant binary outcomes: (1) selection of collagen-based products for moderate-sized perforations, and (2) same-day repair versus repair at a later time. Models were fitted for each key predictor (number of patients seen, number of sinus perforation cases managed, years in practice, self-rated confidence, specialization, and practice location), while controlling for age, sex, and highest education level. Adjusted odds ratios (aORs) with 95% confidence intervals (CIs) were reported, and ordinal predictors were modeled per one-category increase. All analyses were performed using two-sided tests, and results were interpreted descriptively given the exploratory, non-interventional nature of the study.

### Ethics

The study protocol received IRB approval in accordance with the Declaration of Helsinki and relevant federal regulations (45 Code of Federal Regulations Part 46). Electronic informed consent preceded survey access. Respondents could skip any question and withdraw at any time without penalty. No identifiable or protected data were collected. All responses were stored in encrypted, password-protected servers, and only the research team had access to the de-identified dataset.

## Results

A total of 193 dental practitioners participated in this survey. The majority of respondents were male (72.8%). Participants represented a broad age range, with the largest proportions falling within the 30–39 years (27.6%) and 40–49 years (27.1%) age groups. With respect to educational background, most respondents possessed advanced qualifications, with 38.9% holding doctoral degrees and 37.8% having completed residency programs. A further 17.1% reported fellowship or specialist training. In terms of specialization, general dentists comprised the largest subgroup (62.2%), followed by oral surgeons (25.4%), periodontists (8.8%), pediatric dentists (2.1%), and endodontists (1.6%) (Table 1).

**Table 1:** Demographic characteristics of the participants

Variables	n	%
Age		
Under 30 years	32	16.7
30–39 years	53	27.6
40–49 years	52	27.1
50–59 years	28	14.6
60 years and above	27	14.1
Sex		
Male	139	72.8
Female	52	27.2
Highest Level of Education		
Other	2	1.0
Bachelor degree	5	2.6
Master’s degree	5	2.6
Doctorate	75	38.9
Residency	73	37.8
Fellowship/Specialist training	33	17.1
Specialization		
Endodontics	3	1.6
General Dentistry	120	62.2
Oral Surgery	49	25.4
Pediatric	4	2.1
Periodontics	17	8.8

### Management practices by perforation size

Management practices for OACs varied considerably according to the size of the sinus perforation. For small perforations (0–2 mm), collagen-based materials with or without suture were the most frequently employed method (53.4%). A notable proportion of respondents (23.3%) opted for no additional treatment. Only 3.1% of practitioners referred these minor defects to specialists (Table 2).

**Table 2:** Frequency and methods of sinus perforation management

Management approach	Small size	Moderate size	Large size
	0–2 mm	2–6 mm	> 7 mm
	n (%)	n (%)	n (%)
Referral to specialist	6 (3.1)	52 (26.9)	105 (54.4)
No additional treatment	45 (23.3)	6 (3.1)	2 (1.0)
Allograft with membrane	3 (1.6)	15 (7.8)	8 (4.1)
Buccal fat advancement	0 (0%)	6 (3.1)	33 (17.1)
Collagen-based material with or without suture	103 (53.4)	60 (31.1)	6 (3.1)
Collagen/bone material with or without suture	21 (10.9)	31 (16.1)	11 (5.7)
Primary closure	5 (2.6)	17 (8.8)	23 (11.9)
Suture only	7 (3.6)	2 (1.0)	0 (0%)
Other methods	3 (1.6)	4 (2.1)	5 (2.6)

As the perforation size increased, treatment patterns shifted toward more interventional or referral-based strategies. For moderate-sized perforations (2–6 mm), the use of collagen-based materials remained common (31.1%), but specialist referral rose substantially to 26.9%. For large perforations (> 7 mm), over half of respondents (54.4%) referred patients to OMS. Among those who undertook repair of the large perforations themselves, buccal fat advancement (17.1%) and primary closure (11.9%) were the most commonly chosen techniques, while collagen-based or bone-composite materials were used infrequently (Table 2).

### Practitioner comfort and confidence

Comfort thresholds for managing sinus perforations differed across dental specialties. General dentists reported the highest comfort managing small perforations up to 2 mm (*n* = 57), with a decreasing number of comfortable handling defects of 3–6 mm (*n* = 46) or 7–10 mm (*n* = 10). In contrast, oral surgeons demonstrated markedly higher confidence, with 45 respondents indicating they were comfortable managing perforations of any size. Periodontists displayed moderate comfort levels, exceeding those of general dentists. Conversely, pediatric dentists and endodontists expressed limited comfort, typically restricting their management to small defects only (Table 3).

**Table 3:** Size comfort threshold for sinus perforation treatment among specialties

Size range	Endodontics	General dentistry	Oral surgery	Pediatric	Periodontics
0–2 mm	2	57	0	4	3
3–6 mm	1	46	1	0	1
7–10 mm	0	10	3	0	4
Any size	0	6	45	0	9

### Factors influencing material and technique selection

When respondents were asked to identify the main factors influencing their choice of materials or products for sinus perforation repair, ease of use emerged as the most frequently reported determinant (33.7%). The second most common factor was alignment with practice preferences or protocols (26.8%), followed by the type of material (24.2%). Longevity of the materials (5.8%) and cost (0.5%) were of minimal influence, while 8.9% of respondents mentioned other factors (Table 4).

**Table 4:** Main reasons for choosing the product for sinus perforation treatment

Variables	N	%
Choice of the practice	51	26.8
Cost	1	0.5
Ease of use	64	33.7
Longevity	11	5.8
Type of material	46	24.2
Other reasons	17	8.9

### Timing of sinus repair

With respect to the timing of sinus repair following perforation, the vast majority of practitioners (80.8%) reported performing the repair on the same day that the sinus communication was detected. A smaller proportion delayed intervention for one to seven days (5.8%), while 2.9% postponed closure for one to two weeks, and 10.5% delayed repair beyond two weeks. Same-day management was therefore the predominant clinical approach (Table 5).

**Table 5:** Time point for repairing the sinus after sinus perforation occurs

Time point	N	%
Same day (day 0)	139	80.8
1–7 days after	10	5.8
8–14 days after	5	2.9
>2 weeks after	18	10.5

### Associations between management decisions and practitioner characteristics

Analyses of associations between management choice and practitioner characteristics (Table 6) revealed several significant patterns. The area of specialization exhibited a strong relationship with management strategy (Cramer’s V = 0.654, *p* < 0.001). The number of sinus perforation cases previously managed also showed a relatively strong relationship with treatment choice (Cramer’s V = 0.403, *p* = 0.007). Moderate associations were identified for age, educational level, number of patients seen, years of dental practice, and self-rated confidence (Cramer’s V ranging 0.279–0.327), suggesting that experience and professional background meaningfully influenced management behavior. Because these univariate associations do not account for overlap among practitioner characteristics (e.g., higher patient volume and higher confidence may co-occur with greater prior perforation experience), we additionally report demographic-adjusted multivariate logistic regression results as the primary basis for interpreting independent associations. In models examining collagen-based product selection for moderate perforations (Table 7), greater experience managing sinus perforations (aOR = 1.49; *p* = 0.006) and higher confidence (aOR = 1.69; *p* = 0.010) were independently associated with increased odds of selecting collagen-based products. In contrast, several variables that showed moderate univariate associations, such as patient volume and years in practice, were not significant after adjustment, suggesting that their univariate signals may reflect confounding by experience- or confidence-related factors rather than independent effects.

**Table 6:** Association between management choice and key variables

Key variables	*p*-value	Cramer’s V	Strength of association
Age	0.105	0.300	Moderate
Sex	0.542	0.066	Negligible
Highest level of education	0.102	0.327	Moderate
Number of patients seen	0.031	0.322	Moderate
Number of sinus perforation cases managed	0.007	0.403	Relatively strong
Year of practice dentistry	0.068	0.320	Moderate
Confidence in managing sinus perforations	0.035	0.279	Moderate
Area of specialization	<0.001	0.654	Strong
Practice location	0.152	0.154	Weak

**Table 7:** Multivariable predictors of collagen-based product selection for moderate perforations, controlling for age, sex and education

Predictor	aOR	95% CI	* p*-value
Number of patients seen*	0.78	0.57–1.07	0.130
Number of sinus perforation cases managed*	1.49	1.12–1.97	0.006
Year of practice dentistry*	1.14	0.69–1.87	0.611
Confidence in managing sinus perforations*	1.69	1.13–2.51	0.010
Area of specialization (oral surgery vs. others)	1.36	0.59–3.12	0.469
Practice location (urban vs. rural)	1.35	0.44–4.13	0.600

*Note: aORs reflect one-category increase.

In contrast, the associations between timing of repair and practitioner characteristics (Table 8) were generally weak or negligible. Although practitioners with greater experience managing sinus perforations tended to favor same-day closure (Cramer’s V = 0.210, *p* = 0.107), this relationship did not reach statistical significance. Other demographic and professional factors, including age, sex, education, and practice location, exhibited only weak to negligible associations with timing decisions (Cramer’s V ≤ 0.179). Consistent with these patterns, demographic-adjusted multivariate models did not identify significant independent predictors of same-day versus delayed repair (Table 9). Practice location (urban vs. rural) showed a non-significant trend toward lower odds of same-day repair (aOR = 0.18; *p* = 0.088).

**Table 8:** Association between time point of repair and key variables

Key variables	*p*-value	Cramer’s V	Strength of association
Age	0.239	0.179	Weak
Sex	0.212	0.096	Negligible
Highest level of education	0.472	0.163	Weak
Number of patients seen	0.745	0.085	Negligible
Number of sinus perforation cases managed	0.107	0.210	Moderate
Year of practice dentistry	0.515	0.138	Weak
Confidence in managing sinus perforations	0.276	0.150	Weak
Area of specialization	0.054	0.147	Weak
Practice location	0.257	0.086	Negligible

**Table 9:** Multivariable predictors of time point of repair, controlling for age, sex and education

Predictor	aOR	95% CI	*p*-value
Number of patients seen*	0.11	0.08–1.58	0.575
Number of sinus perforation cases managed*	1.00	0.76–1.34	0.976
Year of practice dentistry*	0.89	0.50–1.59	0.698
Confidence in managing sinus perforations*	1.01	0.66–1.55	0.950
Area of specialization (oral surgery vs. others)	1.17	0.48–2.85	0.731
Practice location (urban vs. rural)	0.18	0.02–1.30	0.088

*Note: aORs reflect one-category increase.

## Discussion

### Overview of findings

This study examined contemporary management strategies, influencing factors, and practitioner confidence levels in the treatment of OACs. Findings revealed a clear, size-dependent pattern of clinical decision-making, reflecting a largely evidence-aligned yet heterogeneous approach across respondents. While most respondents displayed an understanding of appropriate management principles, there was notable variation in comfort thresholds and material selection across specialties, underscoring the influence of clinical experience and postgraduate training on decision-making.

Inferential analysis confirmed these trends, with specialization showing the strongest association with management approach, underscoring that surgical training remains the most critical determinant of treatment choice. The demographic profile, comprising predominantly mid-career dentists with advanced qualifications, suggests that OAC management is primarily undertaken by clinicians possessing substantial clinical maturity and procedural exposure. The predominance of general dentists and oral surgeons among respondents reflects clinical reality: OACs typically arise during extractions performed by general practitioners [19], but often require surgical expertise for definitive closure [20]. This cohort distribution thus offers insight into real-world management practices across varying levels of training and clinical responsibility among respondents.

### Management patterns by perforation size

The study demonstrated a progressive escalation in management complexity corresponding to perforation size. For small perforations (0–2 mm), the predominant use of collagen-based materials with or without suturing reflects a conservative, biologically favorable approach [21]. This practice is consistent with the literature, reporting high success rates using platelet-rich fibrin and collagen-based materials for small perforations, attributing favorable outcomes to their biocompatibility and ability to promote epithelial closure [22, 23]. The decision by nearly a quarter of respondents to forego additional intervention further supports the notion that very small sinus openings often close spontaneously if proper sinus precautions are maintained [24].

For moderate perforations (2–6 mm), a shift toward more interventional strategies was evident. Collagen-based and bone grafting materials remained popular, but the rise in specialist referrals suggests practitioners’ recognition of the increased risk of chronic communication and sinus pathology at this defect size. These findings align with the literature, emphasizing multidisciplinary management for larger or infected OACs involving both dental and otolaryngologic specialists [25].

In cases of large perforations (> 7 mm), more than half of respondents referred patients to OMS. Among those who performed closure themselves, buccal fat pad advancement and primary closure were the predominant techniques. This trend is consistent with the classic surgical principles demonstrating the reliability of buccal advancement flaps and buccal fat pad grafts for large oroantral defects [26, 27]. Collectively, these results indicate that practitioners tailor interventions according to the size and complexity of the defect, consistent with accepted surgical protocols, though without formal standardization. The strong statistical relationship between specialization and management choice (Cramer’s V = 0.654) further reinforces these clinical trends: oral surgeons, with extensive experience, were significantly more likely to perform advanced surgical procedures than other dental practitioners [28]. This finding underscores how procedural scope and surgical competence directly shape management strategies in real-world settings.

### Specialty-Based differences in comfort and competence

The study highlighted significant variation in comfort thresholds across dental specialties. Oral surgeons reported the highest confidence in managing OACs of any size, followed by periodontists, while general dentists and other specialists exhibited reduced comfort with moderate or large perforations. These differences mirror the structure of postgraduate training, where surgical exposure and procedural repetition build familiarity and technical skill [29]. This study’s findings further supported this observation, with specialization demonstrating the strongest association with management approach, and only moderate links between confidence level and decision-making (Cramer’s V = 0.279). The data therefore suggest that while clinical confidence influences management choices to a degree, structured surgical training remains the more decisive factor.

These differences highlight an educational gap within general dental curricula, where instruction on surgical management of OACs is often limited. Given that general dentists frequently encounter small perforations during extractions, targeted training in recognition and immediate management could enhance competence and reduce delays in appropriate intervention. Continuing education courses emphasizing surgical anatomy, flap design, and closure techniques would be especially valuable in bridging this skill gap.

### Determinants of material and technique selection

Ease of use emerged as the most influential factor guiding the selection of closure materials and techniques, surpassing considerations such as longevity or cost. This finding reflects a pragmatic approach to clinical decision-making, wherein practitioners prioritize materials that are predictable, easy to handle, and compatible with their existing workflow. However, this pattern also suggests a potential disconnect between ideal evidence-based material selection and real-world decision-making. While ease of handling may reflect workflow efficiency, clinician familiarity, and perceived predictability, it may not consistently align with selection based on comparative success rates, durability, or patient-centered outcomes. Accordingly, although many respondents demonstrated size-appropriate management patterns (e.g., escalation to flap-based closure or referral for larger defects), material selection within defect categories may be influenced more by convenience and availability than by outcome-driven evidence. This reinforces the need for standardized, outcomes-informed guidance and training that translate the existing literature into practical, technique- and product-specific recommendations. Similar findings have been reported in studies examining biomaterial selection for bone grafting and socket preservation, where clinician familiarity and handling characteristics were key determinants of choice [30].

The relatively low emphasis on cost may indicate that economic considerations are less influential in this context; however, the predominance of ‘ease of use’ suggests that convenience, familiarity, and workflow considerations may often outweigh durability- or outcomes-based criteria in material selection. Nonetheless, the finding that “practice preference” and “type of material” were also significant determinants highlights the influence of habit and institutional culture in shaping treatment practices. Such variability further reinforces the need for standardized, evidence-based guidelines to ensure consistency and optimal outcomes across clinical settings.

The moderate association between management approach and education level (Cramer’s V = 0.327) suggests that advanced academic training may encourage evidence-based material selection and awareness of technique-specific advantages. However, since ease of use remained the most reported factor, practical experience and material handling familiarity appear to outweigh purely academic influences in real-world decision-making. In multivariate analyses focused on moderate perforations, experience managing sinus perforations and self-rated confidence emerged as the key independent correlates of collagen-based product selection, whereas variables that appeared relevant in univariate testing (e.g., patient volume and years in practice) were no longer significant. This pattern suggests that apparent univariate relationships may be driven by underlying clinical exposure and perceived comfort rather than by broader practice characteristics themselves.

### Timing of sinus repair

An overwhelming majority of practitioners (80.8%) reported performing sinus repair on the same day that the perforation was identified, indicating strong adherence to evidence-based principles advocating early intervention. Early closure minimizes bacterial contamination, prevents epithelialization of the tract, and reduces the risk of chronic oroantral fistula formation [21]. The findings showed that the timing of sinus repair was not significantly associated with most practitioner variables, including age, education, or specialty.

The weak to negligible Cramer’s V values (≤ 0.179) suggest that immediate closure has become a widely accepted norm across the dental community, transcending demographic or experiential differences. The only variable showing a moderate, though non-significant trend was the number of sinus perforations previously managed, indicating that clinicians with greater case exposure may be slightly more likely to intervene promptly. These findings suggest strong consensus regarding the importance of same-day repair, reflecting uniform adherence to contemporary clinical standards. Reinforcing this interpretation, multivariate regression did not identify any practitioner characteristic as an independent predictor of same-day repair. Taken together, the multivariate results suggest that timing decisions are relatively uniform across respondent subgroups, and that univariate differences, when present, should be interpreted cautiously.

### Limitations

Several limitations warrant consideration. The study employed a cross-sectional, self-reported survey design, which is inherently subject to recall bias and social desirability bias; practitioners may have overestimated their adherence to best practices or underreported less common approaches. In addition, because the survey was distributed through multiple channels, including social media, the number of practitioners who viewed the invitation (and the number of unique individuals reached) could not be determined. As a result, an overall response rate could not be calculated, limiting formal assessment of non-response bias. This introduces the possibility of self-selection, whereby clinicians with greater interest in OAC management or higher confidence may have been more likely to participate, potentially inflating reported comfort levels and the apparent frequency of evidence-aligned practices. Additionally, self-reported comfort is a subjective measure and may not correlate with clinical competence or treatment success. The potential for confidence–competence mismatch (including a Dunning–Kruger effect, wherein individuals with lower expertise may overestimate their ability) represents a further source of bias when interpreting comfort thresholds and self-assessed management capability. The survey also categorized “collagen-based materials” as a single response option, without distinguishing hemostatic collagen plugs/sponges (e.g., CollaPlug-type materials) from collagen barrier membranes used for guided bone regeneration. Because these products may have different indications in OAC management, this grouping may have obscured technique-specific decision-making.

Generalizability is also limited. The sample was obtained through convenience sampling, and the size and geographic distribution may not fully represent all U.S. practice settings. Differences in training curricula, clinical exposure, and resource availability across regions or institutions may influence management decisions and were not examined in depth. Accordingly, findings should be interpreted as describing patterns among survey respondents rather than providing nationally representative estimates for the U.S. dental workforce.

Some specialty groups were represented by small numbers (e.g., pediatric dentistry and endodontics) and may have relatively limited exposure to OACs; therefore, these subgroup results should be interpreted descriptively rather than as definitive specialty-level estimates. Relatedly, the specialty distribution was imbalanced, with general dentists comprising the majority, which may have shifted overall responses toward more conservative management approaches and reduced the stability of comparisons involving smaller subgroups.

Finally, this study assessed reported practices rather than clinical outcomes. The findings describe management preferences and perceived confidence but cannot determine comparative effectiveness or success rates of specific techniques. The survey also did not capture certain potentially important contextual factors (e.g., access to surgical facilities, institutional protocols, or prior complication experience), which may influence decision-making and should be considered in future research.

### Implications for clinical practice and education

Collectively, these findings suggest that practitioners generally apply size-appropriate strategies for OAC management, but that material selection is frequently driven by pragmatic considerations (e.g., ease of use), contributing to variability in practice. However, because confidence was self-reported, educational interventions should prioritize objective skill development and decision support rather than relying on perceived comfort alone. The study findings affirm that specialization exerts the greatest influence on treatment selection, whereas experience-related variables have moderate effects and demographic factors only minimal ones. This underscores the importance of structured surgical training and ongoing education to standardize management practices across dental disciplines.

Integrating OAC management modules into both predoctoral and continuing education programs could reduce disparities in comfort and confidence levels. Exposure to surgical simulation, flap-based closure techniques, and biomaterial handling workshops can further enhance practitioners’ ability to manage OACs effectively. Standardized clinical guidelines, informed by empirical evidence and multidisciplinary consensus, would provide a framework for consistent and optimal patient care across practice settings.

## Conclusion

This study provides a descriptive overview of current OAC management practices among survey respondents practicing in the U.S. Overall, clinicians reported size-dependent escalation in management, with more conservative approaches used for smaller defects and increasing use of referral or flap-based techniques for larger perforations. Experience-related factors, particularly prior management of sinus perforations and self-rated confidence, are more closely tied to material selection, whereas timing of repair appears relatively consistent across practitioner characteristics.

Taken together, these findings indicate that while core management principles are broadly applied, variability persists in technique and product selection. Standardized, outcomes-informed guidance and targeted training may help improve consistency, support appropriate decision-making across practice settings, and optimize patient outcomes.

### Data availability

All data supporting the findings of this study are available within the paper and its Supplementary Information.

## Data Availability

All data supporting the findings of this study are available within the paper and its Supplementary Information.
