# Correction to: Management strategies and determinants of clinical decision-making in oroantral communication among dental practitioners

**DOI:** 10.1007/s10006-026-01527-7

**Published:** 2026-02-13

**Authors:** Man Hung, Samantha Lee, Jacob Marx, Corban Ward, Owen Cohen, Madeleine Tucker, Charles Miller

**Affiliations:** 1 College of Dental Medicine, Roseman University of Health Sciences, South Jordan, UT USA; 2 Department of Family and Preventive Medicine, University of Utah, Salt Lake City, UT USA; 3 Department of Educational Psychology, University of Utah, Salt Lake City, UT USA; 4 Huntsman Cancer Institute, Salt Lake City, UT USA

**Correction to: Oral and Maxillofacial Surgery**

10.1007/s10006-026-01508-w

In the original version of the manuscript, extra digits and typos were included in Tables 7 and 9.

The correct Tables 7 and 9 are shown as below.

Incorrect Table 7

Table 7 Multivariable predictors of collagen-based product selection for moderate perforations, controlling for age, sex and education.

**Table:** 

Predictor	aOR	95% CI	*p*-value	
Number of patients seen*	0.78	0.57–1.07.57.07	0.130	
Number of sinus perforation cases managed*	1.49	1.12–1.97.12.97	0.006	
Year of practice dentistry*	1.14	0.69–1.87.69.87	0.611	
Confidence in managing sinus perforations*	1.69	1.13–2.51.13.51	0.010	
Area of specialization (oral surgery vs. others)	1.36	0.59–3.12.59.12	0.469	
Practice location (urban vs. rural)	1.35	0.44–4.13.44.13	0.600	

*Note: oORs reflect one-category increase

Correct Table 7

Table 7 Multivariable predictors of collagen-based product selection for moderate perforations, controlling for age, sex and education.

**Table:** 

Predictor	aOR	95% CI	*p*-value	
Number of patients seen*	0.78	0.57-1.07	0.130	
Number of sinus perforation cases managed*	1.49	1.12-1.97	0.006	
Year of practice dentistry*	1.14	0.69-1.87	0.611	
Confidence in managing sinus perforations*	1.69	1.13-2.51	0.010	
Area of specialization (oral surgery vs. others)	1.36	0.59-3.12	0.469	
Practice location (urban vs. rural)	1.35	0.44-4.13	0.600	

*Note: aORs reflect one-category increase

Incorrect Table 9

Table 9 Multivariable predictors of time point of repair, controlling for age, sex and education.

**Table:** 

Predictor	aOR	95% CI	*p*-value	
Number of patients seen*	0.11	0.78–1.58.78.58	0.575	
Number of sinus perforation cases managed*	1.00	0.76–1.34.76.34	0.976	
Year of practice dentistry*	0.89	0.50–1.59.50.59	0.698	
Confidence in managing sinus perforations*	1.01	0.66–1.55.66.55	0.950	
Area of specialization (oral surgery vs. others)	1.17	0.48–2.85.48.85	0.731	
Practice location (urban vs. rural)	0.18	0.02–1.30.02.30	0.088	

*Note: oORs reflect one-category increase

Correct Table 9

Table 9 Multivariable predictors of time point of repair, controlling for age, sex and education.

**Table:** 

Predictor	aOR	95% CI	*p*-value	
Number of patients seen*	0.11	0.08-1.58	0.575	
Number of sinus perforation cases managed*	1.00	0.76-1.34	0.976	
Year of practice dentistry*	0.89	0.50-1.59	0.698	
Confidence in managing sinus perforations*	1.01	0.66-1.55	0.950	
Area of specialization (oral surgery vs. others)	1.17	0.48-2.85	0.731	
Practice location (urban vs. rural)	0.18	0.02–1.30	0.088	

*Note: aORs reflect one-category increase

The original article has been corrected.

